# Serum MMP-8 and TIMP-1 concentrations in a population-based cohort: effects of age, gender, and health status

**DOI:** 10.3389/fdmed.2024.1315596

**Published:** 2024-04-04

**Authors:** Julia Ranta, Aki S. Havulinna, Taina Tervahartiala, Katriina Niemi, Ghazal Aarabi, Terhi Vihervaara, Veikko Salomaa, Timo Sorsa, Pirkko J. Pussinen, Aino Salminen

**Affiliations:** ^1^ Oral and Maxillofacial Diseases, Helsinki University, Helsinki, Finland; ^2^ Finnish Institute for Health and Welfare, Helsinki, Finland; ^3^ Institute for Molecular Medicine Finland, FIMM-HiLIFE, Helsinki, Finland; ^4^ Department of Periodontics, Preventive and Restorative Dentistry, Center for Dental and Oral Medicine, University Medical Center Hamburg-Eppendorf, Hamburg, Germany; ^5^ Institute of Dentistry, University of Eastern Finland, Kuopio, Finland

**Keywords:** PMNL collagenase, neutrophil collagenase, biomarker, risk factor, coronary artery disease, circulating

## Abstract

**Background:**

Matrix-degrading proteinases and their regulators, such as matrix metalloproteinase 8 (MMP-8) and tissue inhibitor of matrix metalloproteinase 1 (TIMP-1), may contribute to various pathological events. Elevated MMP-8 concentrations have been associated with e.g., cardiovascular diseases and periodontitis. However, there is little knowledge on the physiological concentrations of these molecules in serum, or the effect of demographic or lifestyle factors on their levels.

**Design and methods:**

We investigated the effect of various demographic characteristics and behavioral habits, such as aging, sex, smoking, and BMI, on serum concentrations of MMP-8 and TIMP-1. We used the FINRISK97 cohort (*n* = 8,446), which has comprehensive information on demographic and lifestyle factors, clinical data, laboratory measurements, and register data available. Further, we investigated the concentrations of MMP-8, TIMP-1, and the MMP-8/TIMP-1 ratio in different age groups of healthy and diseased participants. A *t*-test was used to compare log-transformed mean levels in different groups and linear regression was used to evaluate the association between MMP-8 and TIMP-1 and selected diseases and background variables.

**Results:**

MMP-8 levels decreased with increasing age in the whole population and for women, while TIMP-1 concentration increased slightly with age for the whole population and both genders separately (*p* for linear trend <0.001). The concentrations of MMP-8 were lower and TIMP-1 higher in men compared to women (*p* < 0.001). Additionally, a significant positive association was found for MMP-8 and smoking, CRP, and an inverse association with obesity and fasting time. For TIMP-1, significant positive associations were found with smoking, CRP and obesity, and an inverse association with prevalent diabetes.

**Conclusion:**

The association of serum MMP-8 and TIMP-1 concentrations with cardiometabolic risk is frequently investigated. MMP-8 levels decrease significantly with age and fasting time. In addition, sex, smoking, and obesity are associated with both MMP-8 and TIMP-1 concentrations. These factors should be carefully considered in epidemiological studies on serum MMP-8 and TIMP-1.

## 1 Introduction

Matrix metalloproteinase 8 (MMP-8), also known as collagenase 2 or neutrophil collagenase, belongs to the family of matrix metalloproteinases (MMPs). The main function of MMPs is to degrade extracellular matrix (ECM) during various physiological stages such as development, repair, and remodeling of tissues. The main substrate of MMP-8 is collagen, but in addition, it has numerous other non-matrix bioactive substrates such as fibrinogen, α1-antitrypsin, proteoglycans, angiotensin, insulin receptor, and different chemokines (1, 2).

MMP-8 is mainly released by neutrophils where it is stored prepacked in secondary granules in its latent preform (1). At the sites of inflammation, MMP-8 is released from neutrophils and activated by bacterial or host-derived proteinases or by oxidative mechanisms. Neutrophils are not the only cell type that can produce MMP-8, even though they might be the most prominent ones. Other cells that are known to express MMP-8 are macrophages, T cells, bronchial, oral, and corneal epithelial cells, endothelial cells, fibroblasts, smooth muscle cells and others, for which de-novo expression can often be induced by inflammation (1). In acute inflammation, neutrophils release large amounts of MMP-8 to the extracellular space by degranulation, whereas in chronic inflammation, the release of MMP-8 from various cells like endothelial cells, macrophages, and smooth muscle cells is induced by prolonged exposure to pro-inflammatory cytokines (3, 4).

Tissue inhibitor of metalloproteinase 1 (TIMP-1) binds to MMPs inhibiting their activity. Four different TIMPs have been identified, which all bind to MMPs in 1:1 stoichiometry (5). The MMP-8/TIMP-1 molar ratio is often used as a measurement for the balance between MMP-8 activity and inhibition. TIMP-1 is on MMP inhibitor, but also an independent growth factor, and elevated levels of TIMP-1 have been found to correlate with poorer prognosis of several cancers and to associate with tumor stage and progression (6). TIMP-1 levels have also been shown to be a powerful predictor for some cardiovascular disease (CVD) outcomes, like myocardial infarction (MI) and stroke, and ultimately cardiovascular death (7–10).

High MMP-8 levels are considered an independent risk factor for CVD like MI, coronary heart disease (CHD), and death (11). Serum MMP-8 concentrations have been directly associated with risk of future CVD, and the concentrations of MMP-8 and TIMP-1 showed a positive correlation with the severity of stroke (9, 12). In coronary artery disease (CAD), MMP-8 plasma concentration was found to be increase as the severity of the disease increased (13). MMP-8 and TIMP-1 have prognostic significance in several cancers, but it has also been reported that MMP-8 has distinct roles in different cancers (1, 14–17). MMP-8 may also contribute to the progression of insulin resistance by cleaving insulin receptors (2). In addition, serum MMP-8 concentration predicted the severity of acute pancreatitis (18).

With this background, the determination of MMP-8 and TIMP-1 concentrations in large populations may have clinical significance in risk assessment and disease prediction. However, the effect of background variables and confounding factors on the concentrations must be known for successful study designs. In the present study, our aim was to study how age, sex, and lifestyle habits affect serum concentrations of MMP-8 and TIMP-1, and the MMP-8/TIMP-1 molar ratio in a population-based prospective cohort. Prevalent CVD, diabetes, and cancer were also considered in the analyses. Our hypothesis was that, in addition to prevalent diseases, demographic and lifestyle factors and fasting affect the serum MMP-8 and TIMP-1 concentrations.

## 2 Materials and methods

### 2.1 Study population

FINRISK97, a population-based cohort study (*n* = 8,446) from Finland, was used for this study. The participants were 25 to 74 years old at the baseline (19). Participants answered extensive questionnaires including questions about smoking habits, alcohol consumption, years of education and doctor-diagnosed diseases, such as diabetes and CVD. Participants were classified as never smokers, if they replied “no” to the question “Have you ever smoked?” Clinical examination was also performed to assess, e.g., height, weight, body mass index (BMI), blood pressure, and waist/hip ratio. Participants with a BMI ≥ 30 kg/m^2^ were classified as obese. Venous blood samples were drawn from the participants for further examinations. Participants were asked to fast for a minimum of 4 h before sampling and to avoid heavy meals beforehand. Prevalent diabetes was defined as doctor-diagnosed disease, and hypertension was defined according to American Heart Association guidelines (20). Healthy individuals were defined as those without CVD, diabetes or cancer.

### 2.2 Laboratory analyses

The baseline measurements included serum C-reactive protein (CRP) and γ-glutamyltransferase (γ-GT) concentrations (21). Serum MMP-8 concentrations were determined by time-resolved immunofluorometric assay (IFMA; Medix Biomedica, Espoo, Finland) and serum TIMP-1 by chemiluminescent micro assay (CMIA; Abbott, Architect *i2000,* Kauniainen, Finland) according to manufacturers' instructions. The interassay coefficient of variations (CV %) for MMP-8 and TIMP-1 were 3.1 and 7.3% (8).

### 2.3 Statistical analyses

Statistical analyses were performed by using SPSS (IBM® SPSS® statistics v.25). Before analyses, MMP-8 and TIMP-1 concentrations were log_10_-transformed as their distributions were clearly skewed in histograms. MMP-8 concentrations were available for 8,349 individuals and TIMP-1 for 7,847 individuals. MMP-8 and TIMP-1 ratio was calculated by dividing MMP-8 molar concentration with TIMP-1 molar concentration. MMP-8/TIMP-1 ratio values were also log_10_-transformed.

**Figure 1:**
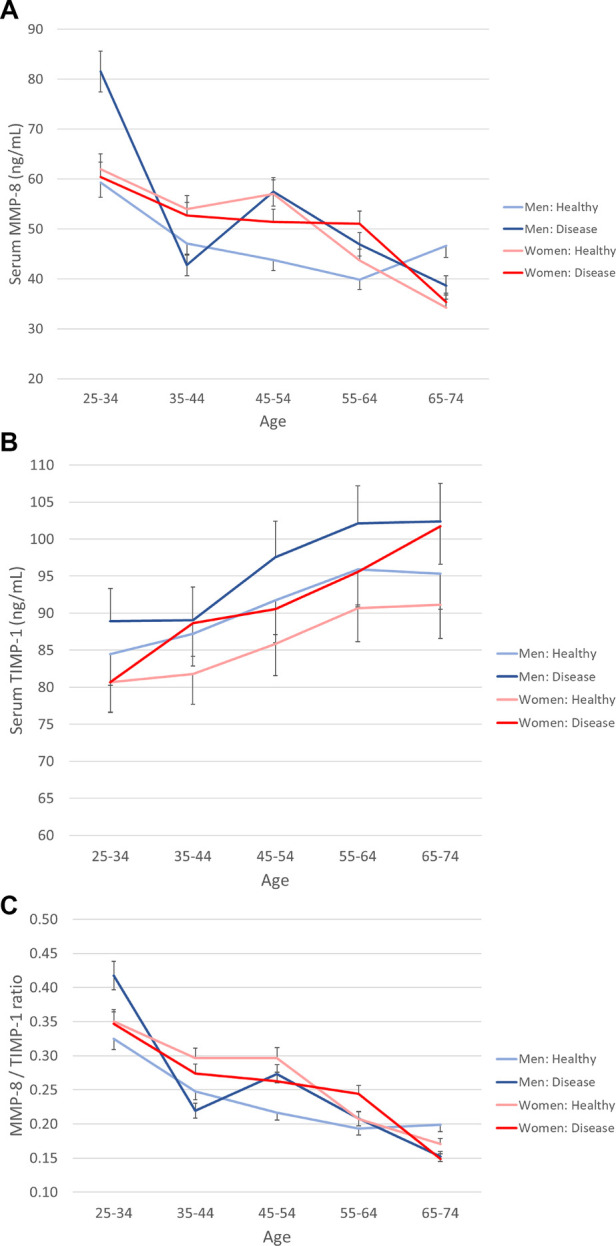
Serum MMP-8, TIMP-1, and their ratio in genders according to age and health status. The population was divided into ten-year age groups and genders are presented separately. Healthy is defined as without prevalent CVD, diabetes, or cancer. (**A**) MMP-8, (**B**) TIMP-1, (**C**) MMP-8/TIMP-1 molar ratio. Error bars represent 95% confidence intervals.

To compare the concentrations between different reference groups and chosen disease groups, *t*-tests were used for the transformed values of MMP-8, TIMP-1, and their ratio. The differences between prevalent disease groups vs. healthy reference groups were calculated separately. In addition, 2.5 and 97.5 percentiles were used to determine the reference intervals in study groups. Furthermore, we analyzed how the concentrations of MMP-8, TIMP-1 and the ratio of MMP-8 and TIMP-1 differ between different age groups in the whole population and in men and women separately. The linear trends with age were tested by linear regression. After analyzing the data, logarithmic values were back-transformed to original concentrations for Table 2, Supplementary Table S1, and Figure 1. The linear regression model was used to analyze the association between MMP-8, TIMP-1 and MMP-8/TIMP-1 concentrations (predicted variables) and age, gender, smoking, hypertension, fasting time, BMI, CRP, and prevalent CVD, diabetes, and cancer (predictor variables). When comparing “healthy” and “disease” groups, “healthy” is defined as without prevalent CVD, diabetes, or cancer. Bivariate Spearman rank correlations were calculated for MMP-8 and TIMP-1 concentrations with fasting time. All tests were two-tailed, and *p* values < 0.05 were considered statistically significant.

## 3 Results

Table 1 displays the characteristics of the FINRISK97 cohort (*n* = 8,446) at the baseline. The mean age of the participants was 49 years, and approximately half of the population were men and half were women.

**Table 1:** Characteristics of the FINRISK97 study population.

	*N* (%)
Gender	
Women	4,140 (49.6)
Men	4,209 (50.4)
Smoking	
Never	4,326 (51.8)
Ex	1,881 (22.5)
Current	1,924 (23.0)
Median (IQR)	
CRP (mg/L)	1.15 (1.9)
ɣ-GT (U/L)	24.0 (21.0)
Alcohol (g/week)^a^	26.6 (79.2)
Mean (SD)	
Age (years)	49.0 (13.5)
25–34	29.8 (2.8)
35–44	39.6 (2.9)
45–54	49.3 (2.8)
55–64	59.4 (2.8)
65–74	69.3 (2.8)
BMI (kg/m^2^)	26.7 (4.6)
Waist/hip	0.87 (0.1)
Education (years)	11.3 (4.0)
Blood pressure (mmHg)	
Systolic	138.0 (20.7)
Diastolic	82.7 (11.6)

SD, standard deviation; IQR, interquartile range; BMI, body mass index; BP, blood pressure; CRP, C-reactive protein; ɣ-GT, gamma-glutamyl transferase.

^a^ 12 g equaling one portion of alcohol.

Medians with 95% confidence intervals for MMP-8, TIMP-1, and the ratio of MMP-8/TIMP-1 concentrations in different reference groups are presented in Table 2. The median concentration of MMP-8 was higher in women than in men (median in men: 27.6 ng/ml, median in women: 29.9 ng/ml, *p* < 0.001), while the concentration of TIMP-1 was lower in women (median in men: 88.5 ng/ml, median in women: 83.4 ng/ml, *p* < 0.001). The molar ratio of MMP-8 and TIMP-1 was again higher for women (median in men: 0.13, median in women 0.15, *p* < 0.001). In current smokers, MMP-8 concentration was significantly higher than in never-smokers (*p* < 0.001), and for the ex-smoker group, TIMP-1 concentration was significantly higher (*p* < 0.001) than in never-smokers.

**Table 2:** Comparison of MMP-8 and TIMP-1 concentrations in chosen disease groups, conditions or lifestyle factors compared to healthy references within the group.

		MMP-8 (ng/ml)			TIMP1 (ng/ml)		MMP-8/TIMP-1 (mol/mol)		
	*n* (%)	Median (95% CI)	*p*	*n* (%)	Median (95% CI)	*p*	*n* (%)	Median (95% CI)	*p*
All	8,349 (100)	28.7 (5.27–230)		7,847 (100)	86.2 (61.2–148)		7,816 (100)	0.14 (0.02–1.22)	
Gender									
Men	4,209 (50.4)	27.6 (5.06–218)	** **	3,948 (50.3)	88.5 (62.6–158)	** **	3,937 (50.4)	0.13 (0.02–1.10)	
Women	4,140 (49.6)	29.9 (5.38–247)	**<0**.**001**	3,899 (49.7)	83.4 (60.0–137)	**<0**.**001**	3,879 (49.6)	0.15 (0.03–1.32)	**<0**.**001**
Smoking									
Never smoked^a^	4,326 (51.8)	27.3 (5.30–222)		4,131 (52.6)	85.7 (60.7–141)		4,116 (52.7)	0.16 (0.02–1.42)	
Ex-smoker	1,881 (22.5)	27.9 (4.92–214)	0.47	1,813 (23.1)	87.7 (62.6–151)	**<0**.**001**	1,806 (23.1)	0.13 (0.02–1.10)	0.07
Current smoker	1,924 (23.0)	32.2 (5.84–258)	**<0**.**001**	1,838 (23.4)	85.1 (61.4–156)	0.20	1,830 (23.4)	0.14 (0.02–1.17)	**<0**.**001**
Hypertension									
No hypertension, no medication^a^	4,124 (49.4)	30.2 (5.26–248)		3,815 (48.6)	81.8 (59.6–136)		3,798 (48.6)	0.16 (0.02–1.32)	
Medication for hypertension	1,353 (16.2)	26.9 (5.38–191)	**<0**.**001**	1,272 (16.2)	95.7 (66.7–157)	**<0**.**001**	1,268 (16.2)	0.11 (0.02–0.96)	**<0**.**001**
Hypertension, no medication	2,860 (34.3)	27.6 (5.17–226)	**<0**.**001**	2,748 (35.0)	88.0 (63.0–152)	**<0**.**001**	2,738 (35.0)	0.13 (0.02–1.15)	**<0**.**001**
Body mass index									
BMI < 30 kg/m^2^	6,572 (78.7)	29.0 (5.24–244)		6,288 (80.1)	84.1 (60.5–141)		6,260 (90.1)	0.15 (0.02–1.29)	
BMI ≥ 30 kg/m^2^	1,617 (19.4)	26.4 (5.16–200)	**<0**.**001**	1,549 (19.7)	94.7 (66.1–168)	**<0**.**001**	1,546 (19.8)	0.12 (0.02–0.99)	**<0**.**001**
Cardiovascular disease									
No prevalent CVD^a^	8,033	28,8 (5.30–235)		7,567	85.7 (61.0–147)		7,538	0.14 (0.02–1.24)	
Prevalent CVD	330 (4.0)	27.2 (4.31–180)	**0**.**004**	280 (3.6)	97.9 (69.8–157)	**<0**.**001**	278 (3.6)	0.11 (0.02–0.86)	**<0**.**001**
Diabetes									
No prevalent diabetes^a^	7,753	28.8 (5.32–233)		7,281	85.5 (61.0–145)		7,251	0.14 (0.02–1.24)	
Prevalent diabetes	596 (7.1)	26.0 (4.84–203)	**0**.**01**	566 (7.2)	95.5 (63.6–170)	**<0**.**001**	565 (7.23)	0.11 (0.01–2.69)	**<0**.**001**
Cancer									
No prevalent cancer^a^	8,167	28.7 (5.27–230)		7,682	86.0 (61.1–148)		7,651	0.14 (0.02–1.22)	
Prevalent cancer	182 (2.2)	29.2 (4.88–276)	0.91	165 (2.1)	93.9 (65.0–152)	**<0**.**001**	165 (2.1)	0.12 (0.02–1.32)	0.06

Medians with 95% CI are presented. *T*-test for log10 transformed concentrations. For variables with several categories, the reference category is marked with an asterisk. Statistically significant *p*-values are bolded.

^a^Reference group.

MMP-8 concentration was lower and TIMP-1 higher in individuals with hypertension and in those with antihypertensive medication when compared to individuals with no measured hypertension and no antihypertensive medication (*p* < 0.001 for both proteins). When comparing body mass indices, non-obese individuals had higher MMP-8 concentrations than obese individuals (median for the non-obese: 29.0 ng/ml, median for the obese 26.4 ng/ml, *p* < 0.001). For TIMP-1, the non-obese had lower median concentration than the obese (non-obese 84.1 ng/ml, obese 94.7 ng/ml, *p* < 0.001).

TIMP-1 concentration was higher in groups of individuals with heart conditions, diabetes, and cancer, when compared to individuals without prevalent diseases (*p* < 0.001, Table 2). MMP-8 concentration was lower in individuals with CVD (*p* = 0.004) and in those with prevalent diabetes (*p* = 0.01, Table 2).

Figure 1 shows the concentrations of MMP-8 and TIMP-1, and the MMP-8/TIMP-1 molar ratio (medians and confidence interval) in different age groups stratified by gender and disease status. The values are presented in Supplementary Table S1. MMP-8 levels decreased with increasing age in the whole population and for women (*p* for linear trend <0.001). The concentration of TIMP-1 increased with age in all cases (*p* for linear trend < 0.001), whereas the molar ratio MMP-8/TIMP-1 decreased with age (*p* for linear trend < 0.001).

**Figure 2:**
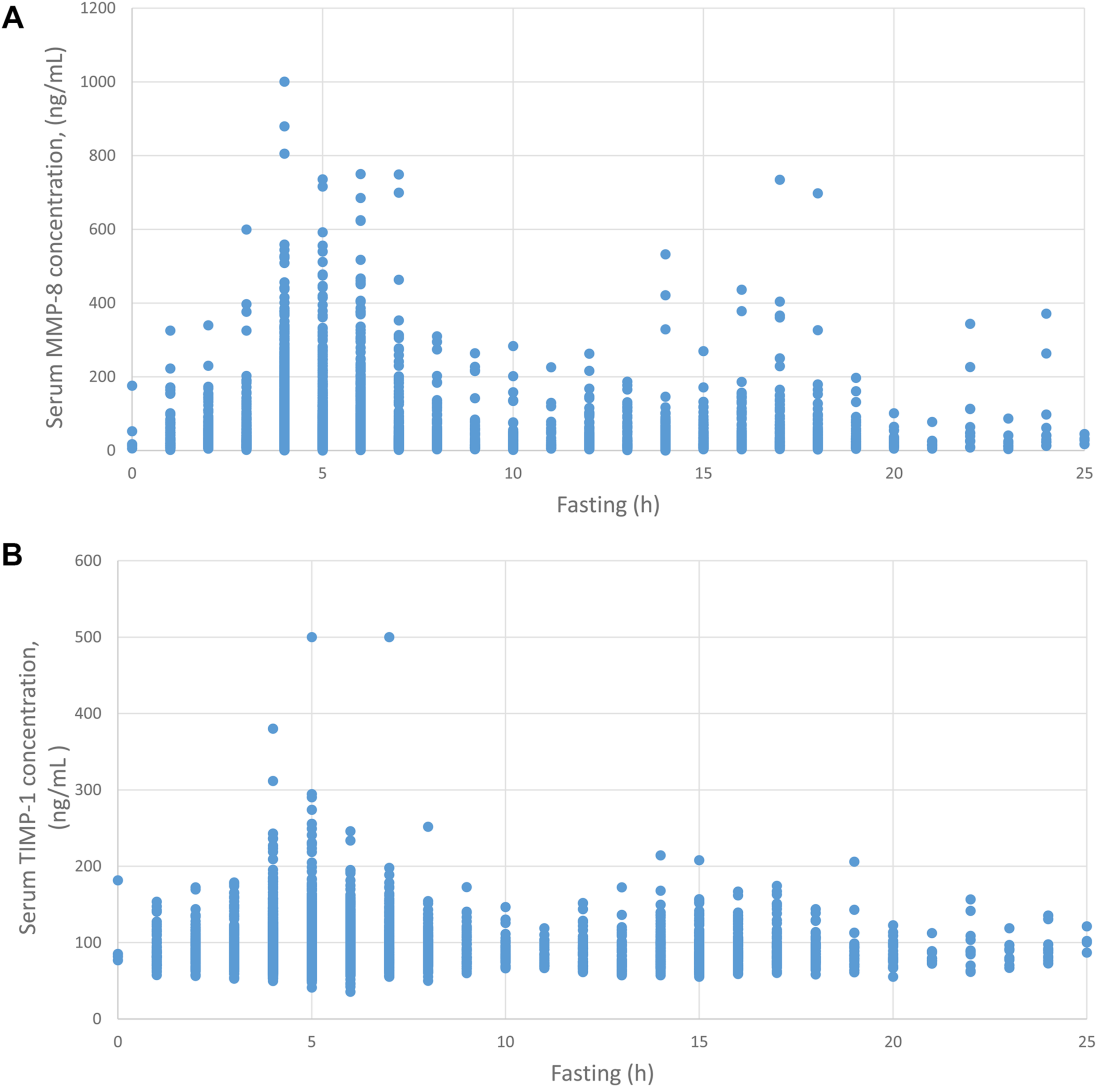
Fasting time and MMP-8 and TIMP-1 concentrations in serum. Serum MMP-8 (**A**) and TIMP-1 (**B**) concentrations are plotted against fasting time before sampling.

Figure 2 displays the concentrations of the proteins and fasting time. Fasting time had a negative correlation with serum MMP-8 concentration (correlation coefficient −0.126, *p* < 0.001). The highest MMP-8 concentrations were measured after 5–8 h of fasting. The longer the fasting, the lower the concentrations of MMP-8 were. Fasting time did not have a significant correlation with TIMP-1 concentration.

The linear regression model was fitted for logarithmically transformed MMP-8, TIMP-1 and the molar ratio of MMP-8/TIMP-1 to determine the association between enzyme concentrations and different parameters displayed in Table 3. For MMP-8, a significant direct association was found with female gender (*p* < 0.001), smoking (*p* < 0.001) and CRP concentration (*p* < 0.001), and significant inverse association with age (*p* < 0.001), fasting time (*p* < 0.001), and BMI (*p* = 0.001). CVD, diabetes, hypertension, and cancer did not associate significantly with MMP-8. Significant direct association with TIMP-1 was found for age (*p* < 0.001), smoking (*p* = 0.042), CRP (*p* < 0.001), BMI (*p* < 0.001), hypertension (*p* < 0.001), and diabetes (*p* < 0.001), and inverse association with female gender (*p* < 0.001).

**Table 3:** Associations between MMP-8, TIMP-1 and MMP-8/TIMP-1 concentrations and selected disease groups, clinical parameters, and lifestyle factors analyzed by multivariable regression.

	MMP-8		TIMP-1		MMP-8/TIMP-1	
	Beta (SD)	*p*	Beta (SD)	*p*	Beta (SD)	*p*
Gender (female)	0.05 (0.010)	**<0** **.** **001**	−0.10 (0.002)	**<0** **.** **001**	0.06 (0.010)	**<0** **.** **001**
Age (years)	−0.13 (0.001)	**<0** **.** **001**	0.21 (0.001)	**<0** **.** **001**	−0.18 (0.001)	**<0** **.** **001**
Smoking (current)	0.06 (0.010)	**<0** **.** **001**	0.02 (0.002)	**0** **.** **042**	0.05 (0.012)	**<0** **.** **001**
CRP (mg/l)	0.18 (0.001)	**<0** **.** **001**	0.16 (0.001)	**<0** **.** **001**	0.14 (0.001)	**<0** **.** **001**
Fasting time (h)	−0.05 (0.001)	**<0** **.** **001**	−0.02 (0.001)	0.063	−0.05 (0.001)	**<0** **.** **001**
Obese	−0.04 (0.001)	**<0** **.** **001**	0.13 (0.001)	**<0** **.** **001**	−0.07 (0.001)	**<0** **.** **001**
Hypertension^a^	0.01 (0.010)	0.48	0.06 (0.002)	**<0** **.** **001**	−0.01 (0.011)	0.68
Prevalent diabetes	0.00 (0.020)	0.99	0.05 (0.004)	**<0** **.** **001**	−0.01 (0.021)	0.32
Prevalent CVD	−0.04 (0.030)	0.69	0.02 (0.006)	0.06	−0.01 (0.027)	0.60
Prevalent cancer	0.02 (0.030)	0.86	0.07 (0.007)	0.53	−0.001 (0.034)	0.91

Linear regression analyses with MMP-8, TIMP-1 or MMP-8/TIMP-1 molar ratio as dependent variables and gender, age, smoking, CRP, fasting time, BMI, hypertension, diabetes, cardiovascular disease, and cancer as independent variables.

^a^ Hypertension was defined as systolic blood pressure >140 mmHg, diastolic blood pressure >90 mmHg, or using regular antihypertensive medication.

Statistically significant *p*-values are bolded.

For the MMP-8/TIMP-1 molar ratio, significant direct associations were found with female gender (*p* < 0.001), smoking (*p* < 0.001), CRP (*p* < 0.001). Inverse associations were found between MMP-8/TIMP-1 ratio and age at baseline (*p* < 0.001), fasting time (*p* < 0.001), and BMI, (*p* < 0.001).

## 4 Discussion

In the present large population-based study, we found that general demographic variables, age and gender, have a significant impact on serum MMP-8 and TIMP-1 concentrations: MMP-8 concentration decreases with age while TIMP-1 increases. This was seen in both genders and in both healthy and diseased subgroups. In a multivariate regression model, MMP-8 was negatively and TIMP-1 positively associated with BMI.

The main source of MMP-8 is degranulating neutrophils (1), which are crucial in the innate immune system. As humans age, their immune system becomes dysregulated, and they become more susceptible to infections. This gradual attenuation of immune function is called immunosenescence. The decline in the adaptive immune system has been thoroughly studied, but less is known about innate immunity and aging. The number of neutrophils does not seem to be affected by age, but several neutrophil functions, including chemotaxis, phagocytic capacity, cytotoxicity, responsiveness to priming, and the ability to respond to survival signals, are reduced with age (22). Attenuated neutrophil function could explain the decrease of MMP-8 levels by age found in our study.

When we compared the non-obese individuals to the obese, we found that obese individuals had significantly lower MMP-8 and higher TIMP-1 concentrations. This is also supported by our linear regression analysis, where BMI had a negative association with MMP-8 and a positive association with TIMP-1. In a previous study, obese women had lower MMP-8 levels, but no significant difference was detected in TIMP-1 concentration when compared to lean women (23). However, a contradictory finding has been reported in a twin cohort on obesity and MMP-8 levels (2), where MMP-8 concentration and the MMP-8/TIMP-1 molar ratio increased with increasing weight. The study population of Lauhio et al. (2) was smaller and younger than our population, which may be the reason for different results.

We found that MMP-8 concentration was significantly higher in smokers than in never-smokers. People who had quit smoking did not differ from the never-smoking reference group. For TIMP-1, no difference was observed between smokers and never-smokers. It has been previously reported that smoking confounds the use of both MMP-8 and TIMP-1 as biomarkers in diagnostics (24) and our results confirm this observation. This could be because smokers are known to have more systemic low grade inflammation.

MMP-8 concentration generally decreased with age. Women had higher MMP-8 levels than men in almost all age groups. The sex differences in MMP-8 concentrations were more apparent in younger age groups, whereas TIMP-1 concentrations presented larger deviations in older age groups. It is known that innate immune responses are stronger in women. Other sex differences are apparent, e.g., in puberty and others throughout life suggesting involvement of both genes and hormones (25). Also contradictory results have been published, since in another large cohort study both MMP-8 and TIMP-1 concentrations were higher in men than women and the sex differences were most pronounced when the women were in their pre-menopausal age (26). Differences may arise from dissimilar samples or methods (27). In the present study, we used serum whose MMP-8 levels are associated with genetic variation of complement factor H (28), an important regulator of the alternative pathway of complement. Furthermore, the alternative pathway activity is lower in women than men (29). Anyhow, these differences contribute to variations in the incidence of several diseases, such as autoimmune diseases, malignancies, and susceptibility to infectious diseases (25).

When we compared patients with CVD or diabetes to the healthy reference group in the whole study population, we found that CVD and diabetes patients had lower MMP-8 levels than the healthy. However, in adjusted regression model, CVD or diabetes were not associated with MMP-8 concentration. The observed difference between CVD patients and healthy therefore probably reflects the fact that CVD patients are older. We found that TIMP-1 concentration was positively associated with hypertension. In previous studies, elevated TIMP-1 levels have been determined as a highly significant, independent predictor for stroke, MI, and cardiovascular death, and to correlate with the severity of stroke (7–9, 11).

MMPs have a prominent role in cancer biology, as they induce the growth of cancerous cells, their differentiation and apoptosis systems, invasion, migration, and tumor angiogenesis (6). In a study on colorectal cancer (CRC), elevated levels of MMP-8 were detected with increasing stage of cancer (30). In the same study, it was found that the more advanced the cancer was, the higher the MMP-8 concentration. For CRC, high MMP-8 and TIMP-1 concentrations were also found to be prognostic (14). Serum MMP-8 and TIMP-1 were prognostic also for hepatocytic carcinoma patients: those with lower concentrations had better overall survival (31). A contrary outcome was seen when studying gastric cancer, where prognosis was worse in patients with low and with high MMP-8 levels when compared to patients with intermediate concentrations (16). In the study of Laitinen et al. (16), high TIMP-1 level was an independent marker for poorer prognosis. MMP-8 is a multifaceted protease with both cancer-promoting and protective facilities (16, 32), and it may have different roles depending on the type of cancer. In our study, MMP-8 was not found to be associated with prevalent cancer, whereas TIMP-1 was. However, we did not have a possibility to analyze different cancer types separately.

Serum MMP-8 levels used in our study were analyzed using immunofluorescence assay (IFMA), which is an established and reliable assay for measuring MMP-8 concentration. ELISA has been also widely used to measure the concentrations, but the results seem to differ markedly between the assays using different capture or detection antibodies (29). Sample material may also explain the conflicting results between different studies. The levels between plasma and serum MMP-8 differ to some extent from each other, even though they have a strong correlation (11, 27).

The strength of our study is the well-characterized and documented large study population. This population enabled us to compare MMP-8 and TIMP-1 levels in different disease groups stratified by age and sex. Even when excluding people that did not meet the healthy criteria, we were left with fairly large reference population. Naturally, more diseases are present in the elderly, which results in a relatively small number of healthy reference individuals in the older age groups.

## 5 Conclusions

According to numerous studies, serum MMP-8 and TIMP-1 concentrations are associated with several diseases and therefore they may have wide clinical significance in both disease risk assessment and prediction. However, when studying the association between MMP-8, TIMP-1 and disease outcomes, statistical models must be carefully adjusted for age, gender, smoking and obesity, as they seem to be major determinants of the systemic concentration of this enzyme and its inhibitor.

## Data Availability

The original contributions presented in the study are included in the article/**Supplementary Material**, further inquiries can be directed to the corresponding author.
