# Supplementary material for: Serum MMP-8 and TIMP-1 concentrations in a population-based cohort: effects of age, gender, and health status
Source: Front Dent Med. 2024 Apr 4;5:1315596. doi: 10.3389/fdmed.2024.1315596 (PMC11797797; doi:10.3389/fdmed.2024.1315596)
Supplement: Supplementary file 1 [file Table1.docx]

Supplementary Material

**stylefixSupplementary Table 1. Comparisons of MMP-8, TIMP-1, and MMP-8/TIMP-1 concentrations in different age groups in whole population and in healthy and diseased groups.**

| **Whole population** | **Age** | **MMP-8 (ng/mL)** | | | **TIMP-1 (ng/mL)** | | **MMP-8/TIMP-1 (mol/mol)** | |
| --- | --- | --- | --- | --- | --- | --- | --- | --- |
|  |  | **n (%)** | | **Median (95 % CI)** | **n (%)** | **Median (95 % CI)** | **n (%)** | **Median (95 % CI)** |
| **All** | **All** | 8349 | | 28.7 (5.27-130) | 7847 | 86.2 (61.2-148) | 7816 | 0.14 (0.02-1.22) |
|  | **25-34** | 1570 (18.8) | | 35.1 (6.36-276) | 1468 (18.7) | 77.8 (58.0-139) | 1459 (18.7) | 0.19 (0.03-1.54) |
|  | **35-44** | 1757 (21.0) | | 30.3 (5.66-212) | 1669 (21.3) | 80.5 (59.9-137) | 1667 (21.3) | 0.16 (0.03-1.22) |
|  | **45-54** | 1828 (21.9) | | 29.6 (4.91-245) | 1762 (22.5) | 86.3 (64.0-146) | 1751 (22.4) | 0.15 (0.03-1.29) |
|  | **55-64** | 1939 (23.2) | | 26.3 (4.91-201) | 1858 (23.7) | 91.4 (66.1-152) | 1851 (23.7) | 0.12 (0.02-0.95) |
|  | **65-74** | 1255 (15.3) | | 23.3 (4.90-178) | 1090 (13.9) | 95.9 (69.0-155) | 1088 (13.9) | 0.1 (0.02-0.79) |
|  |  |  | | **p < 0.001** |  | **p < 0.001** |  | **p < 0.001** |
| **Healthy** | **All** | 7430 | | 28.8 (5.33-236) | 7004 | 85.0 (60.8-145) | 6976 | 0.14 (0.02-1.24) |
|  | **25-34** | 1541 (20.7) | | 35.0 (6.32-276) | 1441 (20.6) | 77.8 (58.0-140) | 1432 (20.5) | 0.19 (0.03-1.52) |
|  | **35-44** | 1684 (22.7) | | 30.1 (5.85-210) | 1600 (22.8) | 80.4 (59.8-137) | 1598 (22.9) | 0.16 (0.03-1.22) |
|  | **45-54** | 1688 (22.7) | | 29.6 (4.97-242) | 1630 (23.3) | 85.6 (63.7-145) | 1620 (23.2) | 0.15 (0.02-1.28) |
|  | **55-64** | 1611 (21.7) | | 26.2 (5.12-195) | 1542 (22.0) | 90.9 (65.7-150) | 1535 (22.0) | 0.12 (0.02-0.95) |
|  | **65-74** | 906 (12.2) | | 23.2 (4.78-182) | 791 (11.3) | 94.5 (68.2-155) | 791 (11.3) | 0.10 (0.02-0.77) |
|  |  |  | | **p < 0.001** |  | **p < 0.001** |  | **p < 0.001** |
| **Disease** | **All** | 919 | | 27.4 (4.78-219) | 843 | 95.6 (66.7-162) | 840 | 0.12 (0.02-0.95) |
|  | **25-34** | 29 (3.2) | | 36.3 (11.5-262) | 27 (3.2) | 76.4 (56.4-109) | 27 (3.2) | 0.22 (0.06-1.49) |
|  | **35-44** | 73 (7.3) | | 33.0 (4.30-235) | 69 (8.2) | 83.8 (62.4-135) | 69 (8.2) | 0.20 (0.02-1.42) |
|  | **45-54** | 140 (15.2) | | 29.4 (4.54-263) | 132 (15.7) | 93.9 (68.4-170) | 131 (15.6) | 0.14 (0.02-0.94) |
|  | **55-64** | 328 (35.6) | | 26.6 (4.82-205) | 316 (37.5) | 95.9 (68.1-161) | 316 (37.6) | 0.12 (0.02-1.11) |
|  | **65-74** | 349 (38.0) | | 23.7 (5.22-173) | 299 (35.5) | 99.5 (71.6-154) | 297 (35.4) | 0.09 (0.02-0.70) |
|  |  |  | **p < 0.001** | |  | **p < 0.001** |  | **p < 0.001** |
| **Women** | **Age** | **MMP-8 (ng/mL)** | | | **TIMP-1 (ng/mL)** | | **MMP-8/TIMP-1 (mol/mol)** | |
|  |  | **n (%)** | **Median (95 % CI)** | | **n (%)** | **Median (95 % CI)** | **n (%)** | **Median (95 % CI)** |
| **All** | **All** | 4140 | 29.9 (5.38-247) | | 3899 | 83.4 (60.0-137) | 3879 | 0.15 (0.03-1.32) |
|  | **25-34** | 855 (20.7) | 37.0 (6.83-273) | | 800 (20.5) | 77.03 (57.2-126) | 794 (20.5) | 0.20 (0.04-1.59) |
|  | **35-44** | 931 (22.5) | 32.2 (5.94-246) | | 885 (22.7) | 79.5 (59.0-126) | 883 (22.8) | 0.18 (0.03-1.32) |
|  | **45-54** | 955 (23.1) | 31.4 (5.60-255) | | 915 (23.5) | 82.9 (62.2-133) | 908 (23.4) | 0.16 (0.03-1.36) |
|  | **55-64** | 980 (23.7) | 26.7 (4.86-196) | | 936 (24.0) | 89.5 (65.5-141) | 931 (24.0) | 0.12 (0.02-0.94) |
|  | **65-74** | 419 (10.1) | 19.0 (4.5-170) | | 363 (9.3) | 92.9 (67.7-155) | 363 (9.4) | 0.08 (0.02-0.8) |
|  |  |  | **p < 0.001** | |  | **p < 0.001** |  | **p < 0.001** |
| **Healthy** | **All** | 3763 | 30.0 (5.52-248) | | 3548 | 82.6 (59.8-133) | 3528 | 0.16 (0.03-1.33) |
|  | **25-34** | 840 (22.3) | 36.9 (6.82-271) | | 785 (22.1) | 77.1 (57.2-126) | 779 (22.1) | 0.20 (0.04-1.57) |
|  | **35-44** | 889 (23.6) | 32.1 (5.99-239) | | 845 (23.8) | 79.4 (58.8-126) | 843 (23.9) | 0.18 (0.03-1.32) |
|  | **45-54** | 881 (23.4) | 31.9 (5.61-255) | | 846 (23.8) | 82.3 (62.2-131) | 839 (23.8) | 0.16 (0.03-1.36) |
|  | **55-64** | 835 (22.2) | 26.6 (5.14-193) | | 798 (22.5) | 89.3 (64.6-137) | 793 (22.5) | 0.12 (0.02-0.95) |
|  | **65-74** | 318 (8.5) | 18.9 (4.44-159) | | 274 (7.7) | 92.2 (67.5-153) | 274 (7.8) | 0.09 (0.02-0.75) |
|  |  |  | **p < 0.001** | |  | **p < 0.001** |  | **p < 0.001** |
| **Disease** | **All** | 377 | 28.4 (4.37-216) | | 351 | 91.2 (64.6-156) | 351 | 0.13 (0.02-0.95) |
|  | **25-34** | 15 (4.0) | 45.3 (16.3-331) | | 15 (4.3) | 76.4 (56.1-99.2) | 15 (4.3) | 0.22 (0.08-1.54) |
|  | **35-44** | 42 (11.1) | 42.3 (4.53-252) | | 40 (11.4) | 82.2 (61.6-133) | 40 (11.4) | 0.21 (0.03-1.47) |
|  | **45-54** | 74 (19.6) | 28.6 (5.71-210) | | 69 (19.7) | 89.6 (64.5-174) | 69 (19.7) | 0.14 (0.02-1.09) |
|  | **55-64** | 145 (38.5) | 29.1 (4.32-203) | | 138 (39.3) | 91.1 (67.8-151) | 138 (39.3) | 0.12 (0.02-0.89) |
|  | **65-74** | 101 (26.8) | 19.0 (5.18-170) | | 89 (25.4) | 97.8 (69.7-170) | 89 (25.4) | 0.08 (0.02-0.78) |
|  |  |  | **p < 0.001** | |  | **p < 0.001** |  | **p < 0.001** |
| **Men** | **Age** | **MMP-8 (ng/mL)** | | | **TIMP-1 (ng/mL)** | | **MMP-8/TIMP-1 (mol/mol)** | |
|  |  | **n (%)** | **Median (95 % CI)** | | **n (%)** | **Median (95 % CI)** | **n (%)** | **Median (95 % CI)** |
| **All** | **All** | 4209 | 27.6 (5.06-219) | | 3948 | 88.5 (62.6-155) | 3937 | 0.13 (0.02-1.11) |
|  | **25-34** | 715 (17.0) | 33.1 (6.18-296) | | 668 (16.9) | 79.0 (59.6-149) | 665 (16.9) | 0.18 (0.03-1.51) |
|  | **35-44** | 826 (19.2) | 28.5 (5.47-186) | | 784 (19.9) | 81.8 (62.1-142) | 784 (19.9) | 0.14 (0.02-1.05) |
|  | **45-54** | 873 (20.7) | 27.5 (4.60-217) | | 847 (21.5) | 88.8 (65.7-156) | 843 (21.4) | 0.13 (0.02-1.06) |
|  | **55-64** | 959 (22.8) | 25.5 (5.28-202) | | 922 (23.4) | 93.5 (67.4-160) | 920 (23.4) | 0.11 (0.02-0.96) |
|  | **65-74** | 836 (19.9) | 25.6 (4.94-180) | | 727 (18.4) | 97.5 (69.6-155) | 725 (18.4) | 0.10 (0.02-0.76) |
|  |  |  | **p = 0.155** | |  | **p < 0.001** |  | **p < 0.001** |
| **Healthy** | **All** | 3667 | 27.7 (5.06-219) | | 3456 | 87.4 (62.2-154) | 3448 | 0.13 (0.02-1.12) |
|  | **25-34** | 701 (19.1) | 33.1 (6.1-297) | | 656 (19.0) | 79.0 (59.5-149) | 653 (18.9) | 0.18 (0.03-1.51) |
|  | **35-44** | 795 (21.7) | 28.8 (5.57-186) | | 755 (21.8) | 81.7 (61.9-142) | 755 (21.9) | 0.14 (0.02-1.05) |
|  | **45-54** | 807 (22.0) | 27.4 (4.61-215) | | 784 (22.7) | 88.3 (65.5-155) | 781 (22.7) | 0.13 (0.02-1.06) |
|  | **55-64** | 776 (21.2) | 25.5 (5.08-199) | | 744 (21.5) | 93.0 (66.9-157) | 742 (21.5) | 0.11 (0.02-0.96) |
|  | **65-74** | 588 (16.0) | 25.1 (4.91-181) | | 517 (15.0) | 95.5 (68.5-155) | 517 (15.0) | 0.11 (0.02-0.77) |
|  |  |  | **p < 0.001** | |  | **p < 0.001** |  | **p < 0.001** |
| **Disease** | **All** | 542 | 27.1 (5.02-221) | | 492 | 97.5 (70.0-164) | 489 | 0.11 (0.02-0.98) |
|  | **25-34** | 14 (2.6) | 33.8 (11.1-196) | | 12 (2.4) | 77.6 (62.8-113) | 12 (2.5) | 0.24 (0.08-1.11) |
|  | **35-44** | 31 (5.7) | 27.7 (4.46-132) | | 29 (5.9) | 86.1 (67.1-141) | 29 (5.9) | 0.11 (0.02-0.75) |
|  | **45-54** | 66 (12.2) | 31.8 (4.60-300) | | 63 (12.8) | 94.8 (73.6-164) | 62 (12.7) | 0.14 (0.02-1.50) |
|  | **55-64** | 183 (33.8) | 25.5 (5.47-211) | | 178 (36.2) | 97.0 (71.0-173) | 178 (36.4) | 0.11 (0.02-0.95) |
|  | **65-74** | 248 (45.8) | 26.2 (5.32-179) | | 210 (42.7) | 99.7 (72.7-152) | 208 (42.5) | 0.10 (0.02-0.70) |
|  |  |  | **p < 0.001** | |  | **p < 0.001** |  | **p < 0.001** |

P for linear trend tested by linear regression is shown.

**
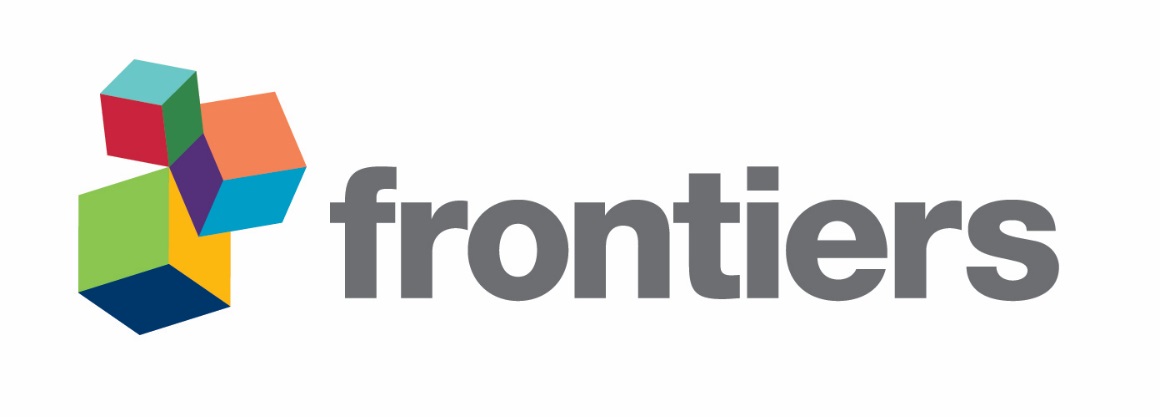
**
